# Multicomponent reactions II

**DOI:** 10.3762/bjoc.10.7

**Published:** 2014-01-09

**Authors:** Thomas J J Müller

**Affiliations:** 1 Heinrich-Heine-Universität Düsseldorf, Institut für Makromolekulare Chemie und Organische Chemie, Lehrstuhl für Organische Chemie, Universitätsstr. 1, 40225 Düsseldorf, Germany

**Keywords:** multicomponent

The concept of multicomponent reactions (MCR) [1] has been around in organic chemistry since the very early days. Indeed, the first example known in literature, the Strecker synthesis of α-aminonitriles [2], has been developed into an industrial process for the production of methionine in an annual scale of several hundreds of thousand tons per year. In addition, a lot of syntheses of heterocycles from the early days are MCR, and these reaction sequences paved the way to a multitude of applications. Moreover, Ugi's groundbreaking developments in isonitrile-based chemistry and his conclusions demonstrated that MCR are not only highly practicable in the light of approaching the ideal synthesis [3–4] as one-pot methodologies, but rely on a reactivity based concept [5]. The perpetual generation of reactive functionalities and reactivity is the underlying general principle. Therefore, MCR are intriguing for industrial applications. But they are also challenging for academia, in particular for the minute and sophisticated fine-tuning of reactivity and selectivity, which is required to concatenate elementary steps to novel sequences.

This Thematic Series on multicomponent reactions is a continuation of the previously released series two years ago [6] and again presents a snap shot of this highly dynamic field. With the traditional formats of letters, full papers, and reviews it spans the broad range of modern chemistry, including organocatalytic, organometallic, transition metal-catalyzed, radical reactions, condensation and isonitrile-based MCR. Biologically active compounds and photonic properties are addressed as well as mechanistic studies and models. The interested reader – whether an expert in the field or a newcomer – will find exciting reports from the current realm of MCR chemistry.

As the guest editor of this Thematic Series I cordially thank all authors for their excellent contributions. I am also grateful to the staff of the Beilstein-Institut for their excellent and professional support.

Thomas J. J. Müller

Düsseldorf, December 2013
